# Copyright as a Barrier to Music Therapy Telehealth Interventions: Qualitative Interview Study

**DOI:** 10.2196/28383

**Published:** 2021-08-13

**Authors:** Amanda Reid, Alex Kresovich

**Affiliations:** 1 Hussman School of Journalism and Media University of North Carolina at Chapel Hill Chapel Hill, NC United States

**Keywords:** telehealth, music therapy, diffusion of innovations, COVID-19, copyright law, fair use

## Abstract

**Background:**

Music therapy is a multifaceted discipline that harnesses the power of music to treat a wide range of patient populations. A therapist who plays music in a private room for a patient is not subject to copyright restrictions on public performances. However, in the wake of the COVID-19 pandemic, music therapy is no longer strictly confined to the face-to-face setting. This study explores music therapists’ perceptions of copyright law with respect to their ability to provide mediated services to their clients.

**Objective:**

The objectives of our study were two-fold. The first was to investigate whether concerns about copyright law are hampering the diffusion of telehealth innovations, and the second was whether these concerns are causing music therapists to avoid therapeutically beneficial telehealth interventions.

**Methods:**

Semistructured interviews were conducted with credentialed music therapists (n=18) in the United States between May 2020 and June 2020. With participants’ consent, we used video conference technology to record and transcribe the in-depth interviews. The median interview length was 45 (SD 16.37) minutes. This theoretically informed study employed thematic analysis of the interview data.

**Results:**

The COVID-19 pandemic accelerated the adoption of telehealth interventions to facilitate therapy outside of private face-to-face environments: environments where music therapy practices are largely shielded from copyright infringement concerns. Five main themes emerged, including therapists’ uncertainty about permissible uses of music and therapists’ erring on the side of caution causing lost opportunities for care. Our interview data suggest music therapists have altered telehealth interventions in suboptimal ways to avoid copyright liability in a physically distanced environment.

**Conclusions:**

Some music therapists “drag their feet” on offering therapeutically appropriate telehealth services to clients because of copyright concerns. Our findings suggest innovative mediated therapies were shied away from or abandoned. These findings offer a novel contribution to the public health literature by highlighting copyright law as an unexpected and unwelcome barrier to the diffusion of music therapy practices in technology-mediated settings.

## Introduction

Music therapy is an evidence-based discipline that uses music to treat a wide range of physiological and psychological conditions and a variety of patient populations [1-10]. The clinical use of musical interventions to achieve therapeutic goals is an increasingly popular telehealth service [11], and the practice benefits millions of youth and adults annually [12]. Telehealth is a vital tool, which is often used to reach underserved patients in rural and urban settings [13]. Preliminary research suggests music therapy via telehealth improves access to care and community music engagement [14,15]. Research also suggests web-based services are promising avenues to increase mental health awareness and treatment options [16,17]. For those practicing via mediated technologies, there are several freely available and fee-based platforms to choose from, including Doxy.me (Doxy.me LLC, Rochester, NY), Microsoft Teams (Microsoft Corporation, Redmond, WA), Skype for Business (Microsoft Corporation), WebEx (Webex by Cisco, Milpitas, CA), and Zoom (Zoom Video Communications Inc, San Jose, CA). These platforms offer real-time video and audio communication between patients and providers [18].

Depending on the client’s needs, a music therapist can create a treatment plan that includes singing, performing, creating, or listening to music. The varied uses of music can include active music listening, song lyric analysis, improvised music playing, and songwriting. It is well established that patient-preferred music yields superior results across various therapeutic interventions [19,20]. Many of the different modes of music therapy intervention involve playing or performing patient-preferred music — music that is often subject to copyright protection [21].

For copyrighted music, rightsholders have several exclusive rights; an exclusive right means the right to exclude others from specified uses of a copyrighted work [22]. Absent an exemption or limitation (eg, fair use [22]), a rightsholder has the exclusive right to make copies of the work, create derivatives of the work, perform the work publicly, and distribute copies of the work publicly. Notably, not all performances of a copyrighted work are proscribed; purely private performances of copyrighted music are not within the rightsholder’s exclusive rights [23]. A legal opinion letter advised the American Music Therapy Association (AMTA) that the use of copyrighted music in face-to-face therapy is not infringing: “A hospital room is private. Hence, one-on-one patient-therapist work within a hospital room is not the kind of setting where performing music or playing music in the form of a sound recording will result in copyright infringement” [24]. In other words, playing copyrighted music is freely permitted when it is not in a public setting. A therapist who plays music in a private room for a patient does not infringe the rightsholder’s exclusive right to perform the work publicly. However, music therapy is no longer strictly confined to the face-to-face setting.

The COVID-19 pandemic catalyzed the rapid adoption of telehealth services to safely deliver care while limiting the risk of exposure to contagion inherent in a face-to-face setting. To facilitate more comprehensive access to physically distanced health care options, the federal government relaxed Health Insurance Portability and Accountability Act (HIPAA) compliance guidelines for telehealth [25]. To further encourage telehealth services, the AMTA issued a statement early in the pandemic clarifying that it “supports the use of telehealth as a means to provide music therapy interventions when beneficial to clients” [26]. Despite the advantages of telehealth, music therapists may be less comfortable using copyrighted music for fear of infringement when using mediated technologies for therapy [27]. The lack of clarity on copyright law in the telehealth space threatens the interventions of those who need physical and mental health care via music therapy. Copyright enforcement efforts can come directly from rightsholders or via automated tools that use algorithms to identify infringing content [28]. The public health literature identifies barriers to real-time telehealth interventions, including a lack of access to basic internet services, inadequate internet speed for audio and video quality, resistance to technology (by patients and providers), and health insurance reimbursement issues [29,30]. However, this literature fails to account for another barrier to telehealth: the threat of copyright enforcement against music therapy interventions that are conducted by mediated communication technologies.

While there is scant literature discussing copyright as a barrier to telehealth, one researcher has captured a snapshot of music therapists’ concerns about copyright. To study telehealth music therapy during the COVID-19 crisis, Carvajal [27] conducted a content analysis of Facebook posts collected from a music therapy group, *Music Therapists Unite*. These posts were collected between January 2020 and April 2020. Three of the music therapists’ posts illustrate uncertainty about the permissibility of therapeutic uses of music under copyright law. One therapist queried the group:

Copyright Question: If I made a recording of myself singing more modern songs and it is a private playlist that only those with the link can see, would that be okay?

Another asked:

I did a Facebook live group called household rhythms where we jammed out using items found around the room to popular songs. I got flagged on Facebook as a copyright [infringer] for one of the songs. It was my understanding if it was for Educational (sic) purposes it was okay to provide recorded music. Can someone offer me some insight as they have asked me to do it again weekly, and I don’t want to get them or myself in trouble.

And lastly, a music therapist asked the Facebook group:

If I wanted to use pre-recorded music for an intervention, can I play that through my phone/speakers at home without getting a copyright strike on Youtube (sic)?

These questions reflect the uncertainty and unease that music therapists have when using copyrighted music to serve clients in virtual spaces. These questions also reflect therapists’ desire to stay out of trouble — copyright trouble either at the hands of rightsholders or algorithmic enforcement.

We hypothesize that such copyright worries may cause music therapists to deviate from preferred treatments. Copyright concerns may not only affect treatment modes but also discourage the delivery of telehealth services altogether. In other words, there is the potential for demonstrable “delays, deformations, and failure to execute mission” [31] in the offering of remote music therapy because of copyright uncertainty. Anxiety and uncertainty about copyright risks further exacerbate existing treatment gaps to those in need [32,33].

According to Rogers’ [34] Diffusion of Innovations theory, an innovation needs wide adoption to self-sustain. And, individuals need clear information about the advantages and disadvantages of adoption. However, the excerpts from Carvajal’s [27] content analysis of the music therapist Facebook group suggest that copyright concerns may hamper telehealth innovations. The early adopters of music therapy telehealth are entering uncharted legal territory. It is unclear whether it is permissible to use copyrighted music in technology-mediated music therapy sessions. We hypothesize that legal uncertainty — rather than uncertainty about telehealth’s practical applications — may be an obstacle to the adoption of this innovation in health care delivery.

## Methods

### Design Strategy

This research adopted a qualitative design using an interpretive description approach, which is appropriate for the study of applied health disciplines [35,36]. Ethical review was performed by the Office of Human Research Ethics at the University of North Carolina at Chapel Hill, and it was determined to be exempt from further review (exemption reference ID 261072). Our interviews were conducted in the United States during the summer of 2020.

The aim of this study was to interrogate the effects of legal uncertainty on the adoption and diffusion of telehealth innovations. Researchers have long recognized that knowledge is contingent upon human practices and social context [37,38]. Meaning is constructed as humans engage with and interpret their sociocultural contexts [39]. The flexibility of interpretive description design permits identification of commonalities of experiences, while also recognizing individual variation [40,41]. The unique experiences of participants are situated within broader patterns of the phenomenon of study [42].

The epistemological perspective of this project views knowledge as socially constructed, and this perspective aligned positively with the phenomena of study. US copyright law is an instrumental doctrine designed to encourage the production and dissemination of creative works.

The lead researcher’s ontological and epistemological assumptions influenced the research strategies of this project. Specifically, the philosophical basis of this study was guided by the lead researcher’s legal training, coupled with her expertise in US copyright law. Consistent with a constructionist perspective, when situating oneself within the research process, it must be acknowledged that a researcher does not approach a study objectively. This reflexivity acknowledges that the lead researcher’s expertise introduced the risk of bias.

To enhance trustworthiness and rigor, our findings were confirmed through 2 methods of triangulation [43,44]. First, participants with a range of experience contributed to the data sources of this study. This data triangulation contributed to a robust and comprehensive understanding of phenomena. Second, an independent communication researcher collaborated in the thematic analysis of the data and provided an unbiased perspective. Both researchers equally contributed to the interpretive thematic analysis of the data. This analytical triangulation contributed independent and credible corroboration of the thematic findings. Moreover, verbatim quotes are included below to offer a clear audit trail of evidence to support these thematic findings.

### Participants

The inclusion criterion was credentialed music therapists working in the United States. Participants were identified using a combination of purposive and snowball sampling approaches. A purposive sampling approach was used to ensure that those recruited represented clinical faculty as well as practitioners and those employed at hospital facilities as well as those self-employed. Participants were initially recruited from a list of music therapists provided to the lead author by a representative at the AMTA. The lead researcher used referrals from these initial interviewees’ networks and then recruited more interviewees via snowball sampling. Finally, some interviewees were recruited using contact information obtained using internet searches for qualified participants. Through these various recruitment and sampling methods, 18 credentialed music therapists met the criterion for an interview.

### Data Collection

All 18 interviews were conducted by the lead researcher between May 19, 2020 and June 19, 2020 and were recorded using Zoom video conferencing. These in-depth, semistructured interview sessions produced over 13 hours of interview data, and each interview averaged 45 minutes in length (SD 16.37 minutes), ranging from 23 minutes to 97 minutes. At the beginning of each interview session, an institutional review board consent script was presented to the participants visually (ie, shared on the computer screen) and aurally (ie, read aloud). Participation was completely voluntary, and consent could be withdrawn at any time. With participants’ consent, the one-on-one interviews were digitally recorded, and the interviews were transcribed by the video conference software. At the end of each interview, participants were offered a US $50 electronic gift card.

The digital recordings and transcripts were uploaded to a secure university-issued computer and then deleted from the video conference platform. The lead researcher anonymized the transcripts, and then a graduate assistant reviewed the transcripts against the audio-only recordings to ensure accuracy. Any questions about grammar or spelling were resolved by the lead researcher. Only the lead author knows the identity of the participants.

Interview sessions began with a topic guide exploring participants’ general experiences as music therapists and the types of therapeutic services they provided. Follow-up questions asked in what particular ways they used music with their clients and why they might use copyrighted music. Participants were then asked how their practices were affected by the COVID-19 pandemic and whether their comfort with using copyrighted music was altered in telehealth settings. An interview schedule initially guided the questioning, and ad hoc follow-up questions were used to further explore salient points.

### Data Analysis

The anonymized and proofed interview transcripts were analyzed using the coding approach by MacQueen et al [45]. Focusing on our hypothesis about the effect of legal uncertainty on telehealth services, we collaborated on codebook development, and we each independently highlighted significant quotes from the interview transcripts [46]. In the coding process, we identified inductive categories through thematic analysis [47]. Through collaboration and iterative refinement, we identified coding patterns reflecting the data’s main themes about telehealth services [48]. Verbatim quotes are included because they richly capture music therapists’ attitudes about copyright. These thick descriptions not only serve to increase conformability in the data analysis process but also reduce the researchers’ influence in the data analysis. Moreover, quotes are attributed to the various participants to illustrate that these emergent themes were consistent from more than one interviewee. With these findings, this study aims to expand the public health literature by revealing an unexpected barrier to the diffusion of innovative telehealth practices.

## Results

### Overview

In total, 18 credentialed music therapists met the criterion for in-depth, semistructured interviews. These participants averaged over 18 years of experience in the field. When asked to self-describe their level of expertise most (14/18, 78%) identified as expert; 3 identified as intermediate (17%), and 1 identified as novice (5%). Most of the participants were female (15/18, 83%). Participants’ varied work settings and client population groups are reflected in Table 1. No other demographic data were solicited from participants.

**Table 1:** Expertise and experience of music therapist (MT) participants (n=18).

MT number	Self-described expertise	Years of MT experience	Work settings and client populations
1	Intermediate	4	Emergency care, intensive care, detox
2	Expert	20	Children
3	Expert	13	Hospice care, cancer care
4	Expert	12	Adolescents from limited-resource communities, mental health
5	Expert	10	Cancer care, dementia care
6	Intermediate	26	Children, adolescents, elder care, mental health
7	Expert	12	Children, adolescents, elder care
8	Intermediate	8	Cancer care, burn care, bereavement
9	Expert	25	Children, mental health
10	Expert	38	Mental health
11	Expert	16	Elder care, hospice care, children, mental health
12	Expert	32	Mental health, cancer care
13	Expert	23	Mental health, substance use disorder, dementia care
14	Novice	>1	Children, adolescents, rehab patients
15	Expert	30	Mental health, elder care, hospice care, children
16	Expert	20	Dementia care, elder care, hospice care
17	Expert	19	Children, adolescents, hospice care
18	Expert	30	Children, adolescents, cancer care

### Thematic Analysis

#### Overview

Analyses of the interview data suggest there are physically distanced clients who are not getting the telehealth services they need — fueled in part by copyright concerns. Our results suggest that risk aversion to copyright liability in technology-mediated interventions is causing some therapists to alter and avoid preferred treatments. Concerns about copyright liability even caused a few therapists to forgo telehealth services altogether because licensing was impractical, unaffordable, or both. Our analysis generated 5 main themes. These themes and representative quotes are listed in Textbox 1.

**Thematic analysis of copyright’s consequences for music therapy telehealth, including representative interview statements for each of the 5 main themes.**

1. Adaptation to COVID-19 pandemic protocols accelerated adoption of technology-mediated interventions:
  “Some of the conversations that I’ve been having with colleagues these days is that the clinical need has changed for the people that we serve, because of the pandemic. If you’re not face-to-face with your folks, then the clinical need has changed.” [MT #5]
  “Teletherapy can also involve downloadables or things that get put on a platform, like YouTube or something like that. And so, the relationship is still happening via email or other platforms of conversation, but the work can happen in these sorts of asynchronous models. And that’s where I think potentially copyright gets involved…” [MT #4]
  “We have had to record and do everything like this [Zoom].” [MT #2]
2. Uncertainty about permissible uses of copyrighted music in technology-mediated interventions:
  “[Music therapists] could also be videotaping, and all of the sudden you’re not just doing live performance, you are talking about potentially a sync license, or mechanical license on top of it. And that gets very complicated very quickly.” [MT #10]
  “For music therapists, it’s kind of a gray area; you’re essentially playing other people’s music and you’re getting paid to provide the service.” [MT #5]
  “Eeek! It [copyright] makes my hair stand on end. I just can’t feel safe. I can’t ever get a straight answer.” [MT #16]
3. Concerns about copyright grew as therapists attempted to adopt technology-mediated interventions:
  “And that was when we really realized like, oh my goodness, there’s a huge gap here, and we don’t know whether we can do this legally using copyrighted music.” [MT #17]
  “As much as I would love to create music therapy sessions and put them on the internet for other people to use, that’s where we run into a lot of copyright issues.” [MT #3]
  “So, as we moved more into creating online content . . . it was so hard to get the answers about what was okay and what was not okay.” [MT #2]
4. Aversion to copyright risks prompted erring on the side of caution:
  “I’ve just been really careful to avoid using previously composed music in this [pandemic] moment.” [MT #3]
  “The problem is there’s just like so many gray areas. It’s hard to determine sometimes what is okay and what is not. And so, I’m always just super cautious about it. I just take a step back and say, I’m not even really going to push that boundary. I’m not going to go there at all, and I’m going to, you know, stay safe over here.” [MT #7]
  “I know one of my colleagues just recently was thinking about [copyright] because she wanted to do a country music sing along on our closed-circuit TV. But she really wasn’t sure if that was appropriate and sort of abandoned the idea.” [MT #8]
  “It’s causing me to not present things that I think could potentially be valuable. So, I might not present the idea of creating a live DJ, or a live performance, because I’m worried about something getting taken down [from YouTube].” [MT #4]
5. Erring on the side of caution triggered lost opportunities of care:
  “That’s absolutely happening… There are practice limitations that are happening. Yeah.” [MT #17]
  “Lack of clarity [on the law] creates those types of environments where I feel like I’m not sure I can suggest something that feels clinically valuable.” [MT #4]
  “Familiar music helps to connect people with their past and with the people that are important to them. We can’t deliver that to them because doing a recording of that would be a violation of copyright law. So, our clients are not getting music that they would have before. They’re not able to access that because of the virus combined with copyright.” [MT #11]

#### Theme 1: Adaptation to COVID-19 Pandemic Protocols Accelerated Adoption of Technology-Mediated Interventions

The COVID-19 pandemic wrought profound changes to therapists’ conventional delivery of music therapy interventions to clients:

COVID put the brakes on face-to-face therapy in outpatient settings.

MT #13

Another therapist said:

I do grief counseling with young children, which has been totally shut down now due to COVID.

MT #8

In sum:

COVID makes it infinitely more complicated because we can’t do the work that we’ve been able to do.

MT #3

The physical distancing protocols prompted by the pandemic forced many outpatient music therapists to shift to mediated platforms to continue to provide services to their clients, as illustrated by this quote:

My preference is to do live as much as possible so that we can interact and I can adapt things in the moment. But in some [rural] places, broadband access isn’t there. So, the live interaction isn’t possible, just because of internet access. And in marginalized communities (in places where they don't have a lot of [broadband] access), recordings are easier to use to have a smooth musical experience — without a lot of freezing and hiccups and things like that.

MT#11

The COVID-19 pandemic forced many outpatient music therapists to shift to mediated platforms to continue to provide services to their clients:

We have had to record and do everything like this [Zoom].

MT #2

Another therapist confirmed the need to shift to mediated services because:

Now, with COVID, I don’t have any face-to-face clients.

MT #16

Nearly all therapists were affected by the pandemic:

Some of the conversations that I’ve been having with colleagues these days is that the clinical need has changed for the people that we serve, because of the pandemic. If you’re not face-to-face with your folks, then the clinical need has changed.

MT #5

Some music therapists reported difficulties as they attempted to adapt to the requirements of physical distancing. In addition to video conference platforms, therapists tried to experiment with alternative methods for delivering therapy, but often with dissatisfying results:

There are so many music therapists who are trying to figure it out, but there isn’t a clear and easy way to do that.

MT #11

For some, therapy continued — albeit in different modes:

Teletherapy can also involve downloadables or things that get put on a platform, like YouTube or something like that. And so, the relationship is still happening via email or other platforms of conversation, but the work can happen in these sorts of asynchronous models. And that’s where I think potentially copyright gets involved…

MT #4

#### Theme 2: Uncertainty About Permissible Uses of Copyrighted Music in Technology-Mediated Interventions

Copyright restrictions were cited as a reason it was not clear and easy to use platform-mediated options for telehealth. In virtual spaces, music therapists might need more than just public performance licenses:

…they could also be videotaping, and all of the sudden you’re not just doing live performance, you are talking about potentially a sync license or mechanical license on top of it. And that gets very complicated very quickly.

MT #10

Frustration at the law’s complexity and uncertainty was expressed by several interview participants. Despite attempts to research copyright law, one therapist perceived fair use to be “incredibly nebulous” [MT #2]. Another therapist concluded that it was ultimately “unknowable” which uses of copyrighted music were safe and which were not [MT #6]. As one therapist stated:

For music therapists, it’s kind of a gray area; you’re essentially playing other people’s music and you’re getting paid to provide the service.

MT #5

Another therapist expressed the uncertainty more colorfully:

Eeek! It [copyright] makes my hair stand on end. I just can’t feel safe. I can’t ever get a straight answer… I’m just flummoxed because it’s so confusing.

MT# 16

In sum, interviewees lacked clarity on which uses of copyrighted music in a technology-mediated delivery format were permissible.

#### Theme 3: Concerns About Copyright Grew as Therapists Attempted to Adopt Technology-Mediated Interventions

It was in the process of exploring technology-mediated therapy options that music therapists reported encountering copyright concerns:

And that was when we really realized like, oh my goodness, there’s a huge gap here, and we don’t know whether we can do this legally using copyrighted music.

MT #17

In virtual spaces, copyright questions were complex and daunting:

Well, we just haven’t been able to devote the time and effort and resources to do that. You can talk to some of these [licensing] companies, but they only deal with certain kinds of licenses. That’s the other complicating factor.

MT#10

Another therapist noted:

As much as I would love to create music therapy sessions and put them on the internet for other people to use, that’s where we run into a lot of copyright issues.

MT #3

“So, as we moved more into creating online content,” one therapist bemoaned that “it was so hard to get the answers about what was okay and what was not okay” [MT #2]. Novel copyright questions were met with unsatisfyingly vague and unclear answers.

In the private, face-to-face settings, therapists uniformly reported feeling comfortable using copyrighted music:

When I’m face to face with the patient I never have said, I’m not going to play that because of copyright. In my mind, I’m not getting paid because I play that music. I’m getting paid for the clinical service.

MT #5

Another therapist emphasized this point:

When I’m face to face with people, I’m not worried about [copyright] at all… Let me be clear, 99.99% of the time I don’t worry about copyright; I think it’s covered by fair use. We’re not monetizing anything, and we’re not performing anything in public.

MT #3

However, when therapy was no longer confined to private face-to-face settings, therapists reported copyright concerns. In response to this uncertainty, some interviewees admitted that they shy away from using copyrighted music in technology-mediated therapy sessions:

I’ve just been really careful to avoid using previously composed music in this [pandemic] moment.

MT #3

When asked if there ever was an instance when a copyrighted song would have been more therapeutically appropriate, but because of copyright concerns, it was not used, an interviewee emphatically answered, “Yes, that has happened” [MT #3]. Therapists reported giving wide berth to avoid potential liability:

The problem is there’s just like so many gray areas. It’s hard to determine sometimes what is okay and what is not. And so, I’m always just super cautious about it. I just take a step back and say, I’m not even really going to push that boundary. I’m not going to go there at all, and I’m going to, you know, stay safe over here.

MT #7

In the face of copyright uncertainty, another therapist said she would avoid “anything that could put you at jeopardy,” and “since you don’t know the answer black and white, go the opposite direction…” [MT #2].

But when therapists entirely avoid copyrighted music, it can be therapeutically problematic:

If all our research says patient-preferred music [yield superior results], and then suddenly we can’t use patient-preferred music because of copyright, then that’s a huge problem.

MT #5

One therapist explained the importance of using patient-preferred music:

The problem with [using non-copyrighted music] is that some people are going to be more engaged if you can find that music that they’re really familiar with — that they’re really comfortable with. And most of our referrals are children that are not doing great. It’s the kids in the hospital that are having a tougher time. So, you’re already talking about a kid that’s scared, that’s anxious, that’s maybe anticipating a poke or some kind of difficult procedure. So, using that familiar music is a way of making that connection — building that rapport — and then helping them to start regulating themselves. Honestly, using copyrighted material for a lot of music therapists is really integral to how they practice.

MT #17

In other words, the inability to use a patient’s favorite song hampers a therapist’s ability to connect and build rapport with the patient.

#### Theme 4: Aversion to Copyright Risks Prompted Erring on the Side of Caution

In response to these concerns, some interviewees admitted that they shied away from using copyrighted music in technology-mediated therapy sessions. One therapist candidly acknowledged, “I’m dragging my feet” on therapeutic activities because of copyright uncertainty [MT #4]. This copyright uncertainty:

…creates a situation where I don’t feel comfortable making therapeutically necessary suggestions that I would otherwise make.

MT #4

Another therapist noted:

I know one of my colleagues just recently was thinking about [copyright] because she wanted to do a country music sing along on our closed-circuit TV. But she really wasn’t sure if that was appropriate and sort of abandoned the idea.

MT #8

Therapists acknowledged that copyright concerns are exacerbating lost opportunities for care:

It’s causing me to not present things that I think could potentially be valuable. So, I might not present the idea of creating a live DJ or a live performance because I’m worried about something getting taken down [from YouTube].

MT #4

Automated algorithmic enforcement on platforms like YouTube and Facebook was identified as a concern. Nevertheless, some therapists openly doubted whether famous musicians would directly bring an enforcement action:

I don’t think Ozzy Osbourne is going to come knock on somebody’s door because you use their song in a heartbeat recording.

MT #5

Another noted:

I cannot imagine a world where a [grieving] family in that situation would get in trouble.

MT # 17

Another therapist said that when she has asked musicians for permission to use songs “just for the therapy session,” “almost always they say yes . . . usually they have said, ‘Go for it. No fees’” [MT #13]. In one instance, a therapist wrote to a musician explaining how the song “My Shot” from the play “Hamilton,” was used in therapy to help “clients reflect on what is their shot, what steps they are taking not to throw it away, and where in their lives to they need to rise up” [MT #13]. And shortly thereafter, the therapist “got a really nice note back from Lin Manuel [Miranda] or his publicist (whoever writes his things) that said, ‘This is awesome. I wish you and your patients the best’” [MT #13]. While therapists did not think most musicians would object to therapeutic uses of their music, it was better to be safe than sorry:

I highly doubt that Taylor Swift would come after a child with cancer. But I suppose that there’s a chance that that could happen, or that she could come after the hospital as well. And I don’t want to get anybody in trouble. I don’t want to have a legal battle for one 30-minute session that we’re doing. So, I’m going to step away from that and not make that a problem in the first place.

MT #7

Despite perceiving a low likelihood of enforcement actions by rightsholders, it was seen as safer to err on the side of caution.

#### Theme 5: Erring on the Side of Caution Fueled Unmet Needs for Therapy

Some therapists acknowledged a demand for therapy that was not being satisfied:

I’m going to say, probably at least 60 clients [in nursing homes] that we would be reaching that we’re not reaching right now because of that [copyright].

MT # 11

The therapist explained the unmet need of nursing home patients — patients who are “basically in solitary confinement” and their “functioning is declining” without social interaction and connection with others [MT #11]. Music therapy could potentially help these patients because therapists find that music helps with “socialization and engagement and staying in the here and now” [MT #11]. However, copyright liability was a reason therapists did not feel comfortable offering recorded music therapy sessions. As the therapist put it:

Familiar music helps to connect people with their past and with the people that are important to them. We can’t deliver that to them because doing a recording of that would be a violation of copyright law. So, our clients are not getting music that they would have before. They’re not able to access that because of the virus combined with copyright.

MT #11

Thus, to avoid copyright concerns, some therapists avoided mediated services altogether — because there were no adequate alternatives to patients’ familiar (and copyrighted) music.

Other therapists confirmed that lost opportunities of care were fueled by copyright concerns:

…lack of clarity [on the law] creates those types of environments where I feel like I’m not sure I can suggest something that feels clinically valuable.

MT #4

When asked if patients had therapeutic needs that were unmet, one interviewee confirmed, “That’s absolutely happening”:

I’ve been telling my staff that we have to honor these things [copyright]. And then they push back. And then I push back. And they push back. And, you know, we’re like making a diamond. So yeah, absolutely. There are practice limitations that are happening. Yeah.

MT #17

In other words, our interviews revealed that supervisors and facility administrators are discouraging certain therapeutic practices because it is unclear whether these practices risk copyright liability. Thus, concerns about copyright liability are manifesting as “practice limitations.” Therapeutic practices can be constrained not only by a therapists’ own concerns but also by a therapist's supervisor’s concerns.

## Discussion

### Principal Findings

Our primary objective was to obtain greater insight into whether copyright concerns affected music therapists’ delivery of computer-mediated therapy. While telehealth services are not new, widespread experimentation with telehealth services was hastened by COVID-19’s physical distancing requirements. The diffusion of telehealth services offers a range of salutary benefits [30,49]. Nevertheless, the sudden shift to mediated interventions caused considerable confusion in the music therapy community. Research suggests therapists scrambled to answer novel questions about telehealth technology affordances, privacy regulations, and billing protocols [27]. In addition to these questions, our interviews identified uncertainty about copyright law as a contributing factor to music therapists’ hesitance to embrace innovative interventions.

A copyright holder’s exclusive rights to public performances, reproductions, and derivatives of copyrighted music present a unique and underexamined barrier for telehealth services. As our findings suggest, copyright law is not typically a concern for music therapists providing private, face-to-face treatment. A copyright holder cannot restrict purely private performances of copyrighted materials [21,23]. But when therapy transitions to remote delivery via mediated technologies, copyright law is not so clear. Exemptions to patent protection have received notable attention during the pandemic, especially by US policymakers [50]. However, new copyright exemptions have received little attention [51]. These findings can help to start those important discussions.

The complexity of US copyright law is reputed to rival the complexity of the US tax code. No court case has evaluated the permissibility of therapeutic uses of copyrighted music. No court case has clarified whether therapeutic uses of music are fair uses of music. Music therapy’s novel uses of copyrighted works put remote delivery of music therapy within a gray zone of US copyright law.

In this gray area of the law, music therapists reported unease. Attempts to research copyright law failed to yield clear, definitive answers, further amplifying music therapists’ confusion. Courts decide fair use questions on a case-by-case basis, and fair use is notoriously difficult to assess in novel settings. As legal scholars note, “The fair use doctrine is famous for its uncertainty” [52].

Not only are questions about fair uses untested in the courts but rightsholders have also failed to offer an efficient mechanism for therapists to license their varied therapeutic uses of music [23]. With neither clear legal precedent on fair use nor an easy way to license their uses, music therapists may be inclined to err on the side of caution to avoid liability. Supporting a risk-averse approach, the AMTA’s COVID-19 Task Force Advisory noted, “A conservative approach simply avoids music covered under copyright in any setting” [53].

### Synthesis of Findings With Prior Work

Emerging research confirms that technology-based music therapy holds tantalizing promise [54]. In the mental health field, the widespread adoption of telehealth has made it the “new normal” [18]. Technology’s perceived usefulness and ease of use are important factors in the successful adoption of new technology [55]. However, the legal dimension to technology’s use has been underexplored in the public health literature.

The value of our work lies in providing new perspectives on barriers to adopting innovative telehealth interventions. The extensive literature on diffusion of innovation has failed to incorporate legal uncertainty as a barrier. Our participants’ thick descriptions offer unique insights on the effects of gray areas of the law.

This study builds on prior inquiry on the effects of copyright law on music therapists. This study draws from a rich corpus of 13 hours of data to answer new research questions, identify distinct themes, and incorporate unique verbatim quotes. Prior research explored how copyright concerns prompt music therapists to act as gatekeepers who discourage patients from sharing therapeutic artifacts on social media [21]. This prior work examined the secondary effects of copyright law, where copyright uncertainty co-opted music therapists to act as proxies for rightsholders. On the other hand, this present work examines the primary effects of copyright uncertainty on music therapists who offer services via telehealth. Rather than discouraging patients’ activities, this study highlights that copyright uncertainty discourages music therapists’ own telehealth activities. We acknowledge the value in sharing health information on social media [56]; however, the results from this study are arguably more urgent because these findings suggest there are lost opportunities of care to those in need.

### Strengths and Limitations

This exploratory study involved 18 music therapists working in the United States. A key strength is the depth of experience of the participants; these participants had over 300 years of combined experience in the field. This study provides an important foundation for exploring music therapists’ views about copyright implications for telehealth. Our findings do not claim generalizability; rather, our aim was a more in-depth understanding of this social phenomenon. To complement the findings in this study, survey analysis should assess a wider pool of music therapists. The views expressed by these participants may not be typical of music therapists. Future research should extend this study through quantitative approaches and should assess how pervasive the concerns identified herein are among a wider sample of music therapists.

Future research should also interrogate whether music therapists in other countries have had similar experiences. Differences in copyright laws in other countries, differences in musical preferences (eg, patient preferences for noncopyrighted or public domain songs), and differences in music therapists’ experiences with telehealth technologies may impair the generalizability of these findings outside of the United States. Moreover, the COVID-19 experience has been unprecedented; it has been generations since the West has experienced a pandemic. Future research should assess how music therapists in other countries adapted during other, recent epidemics, like SARS (severe acute respiratory syndrome) [57] and MERS (Middle East respiratory syndrome) [58]. Future research should assess whether concerns highlighted herein are isolated to Western societies with copyright laws similar to the United States.

Other limitations also exist. The ratio of female to male participants was high, with only 3 of 18 (17%) participants being male. Novice therapists were underrepresented, with only 1 of 18 (6%) self-describing as novice. Lastly, demographic characteristics of the interviewees (eg, age, ethnicity, and religion) were not collected. These limitations in the data are acknowledged.

It is also acknowledged that the lead researcher’s prior research on copyright law had the potential to bias the data analysis. To mitigate these risks, trustworthiness measures were adopted, including collaboration with an independent researcher and inclusion of extensive verbatim quotes. The lead researcher’s law training, while a potential limitation, positively contributed to the quality of the interview data; it enabled thoughtful and appropriate follow-up questions in the semistructured interviews. This exploratory project treads novel ground with music therapists and how copyright concerns are a barrier to telehealth innovations. The quality of these data provides valuable insights not only about music therapists but also may offer insights about other professions that incorporate copyrighted works, like filmmakers [59] or remixers [60,61].

### Conclusions

The lack of clarity on copyright law in the telehealth space has undermined the robust diffusion of therapeutic uses of music. This gray area of the law has prompted some therapists to err on the side of caution. This avoidance is suboptimal in the wake of the shift to technology-mediated interventions and calls for expanded access to underserved populations. Our in-depth interviews confirmed that some music therapists were indeed eschewing their traditional practice modes when using new telehealth innovations because of copyright restrictions. To address the unmet needs for therapy, Congress could amend the copyright statute to include a statutory exemption for therapeutic uses of music [23].

Our interviews revealed that music therapists’ risk aversion has served as a barrier to the diffusion of much-needed telehealth services. These findings offer a unique contribution to the public health literature by (1) highlighting copyright law as an impediment in a technology-mediated setting and (2) revealing there are lost opportunities of care because music therapists are concerned about copyright liability. Thus, legal uncertainty is an underappreciated and unwelcome barrier to the diffusion of telehealth innovations.

## Abbreviations

AMTA: American Music Therapy Association
HIPAA: Health Insurance Portability and Accountability Act
MERS: Middle East respiratory syndrome
SARS: severe acute respiratory syndrome
